# Detection of avian influenza virus in the alien invasive African sacred ibis (*Threskiornis aethiopicus*) in Italy

**DOI:** 10.3389/fvets.2025.1661089

**Published:** 2025-09-08

**Authors:** Alessia Mariacher, Matteo Riccardo Di Nicola, Matteo Senese, Francesco Mariottini, Michela Maestrini, Federica Bellagamba, Carla Donnini, Alessio Capecci, Angela Salomoni, Maria Varotto, Calogero Terregino, Antonella Cersini, Maria Teresa Scicluna

**Affiliations:** ^1^Istituto Zooprofilattico Sperimentale del Lazio e della Toscana “M. Aleandri”, Grosseto, Italy; ^2^Istituto Zooprofilattico Sperimentale del Piemonte Liguria e Valle d’Aosta, Torino, Italy; ^3^Istituto Zooprofilattico Sperimentale del Lazio e della Toscana “M. Aleandri”, Pisa, Italy; ^4^Istituto Zooprofilattico Sperimentale del Lazio e della Toscana “M. Aleandri”, Arezzo, Italy; ^5^Regione Toscana, Firenze, Italy; ^6^Istituto Zooprofilattico Sperimentale delle Venezie, Legnaro, Italy; ^7^Istituto Zooprofilattico Sperimentale del Lazio e della Toscana “M. Aleandri”, Roma, Italy

**Keywords:** AIV, alien invasive species, H5N2, LPAIV, Pelecaniformes

## Abstract

The African sacred ibis (*Threskiornis aethiopicus*), a non-native bird species in Europe, has rapidly expanded its range in Italy, prompting the adoption of national control measures due to ecological and epidemiological concerns. As part of this management plan, 20 ibises were culled in February 2025 in Tuscany (Central Italy), and tested for pathogens relevant to wildlife and public health. RT-PCR and molecular analyses on tracheal and cloacal swabs, revealed the presence of low pathogenic avian influenza virus (LPAIV) subtype H5N2 in 1 out of 20 specimens. Phylogenetic analysis showed that the virus was closely related to recent European LPAIV strains, with the PA gene segment clustered with Asian and Russian isolates from 2021–2022. Two mammalian adaptation markers (S155N and T156A) were identified in the HA protein. Although the detected strain poses minimal zoonotic risk, its presence in a highly adaptable invasive species, raises concerns about the potential role of *T. aethiopicus* as a bridge host in avian influenza transmission cycles. Given the increasing overlap between this species and poultry farming areas, and its scavenging behavior, continued surveillance is essential to assess its epidemiological role. Targeted control actions may be crucial in preventing the establishment of novel wildlife reservoirs and limiting viral evolution towards highly pathogenic forms. Surveillance of alien invasive species should be integrated into broader avian influenza monitoring strategies to protect public health and agricultural biosecurity.

## 1 Introduction

Avian influenza (AI) is a zoonotic infectious disease that affects many domestic and wild bird species, with severe economic impact on the poultry industry due to its high morbidity and mortality rates (1). Wild waterfowl, particularly Anseriformes (ducks, geese, swans) and Charadriiformes (gulls, terns, shorebirds), are recognized as the primary natural reservoirs of influenza A viruses. Nearly all HA and NA subtype combinations have been isolated from these taxa (2). Avian influenza viruses (AIV) are shed via respiratory secretions, feces and saliva, with transmission mainly by aerosols and the fecal-oral route. Migratory movements spread virus along flyways through fecal deposition at stopover and wintering sites, enabling long-distance dissemination (3). Clinical manifestations vary with viral strain and host species; highly pathogenic avian influenza (HPAI) strains cause rapid onset of severe disease and high mortality in poultry, whereas low pathogenic avian influenza (LPAI) strains typically result in mild or subclinical infections (1).

In wild birds, AIV infections are commonly subclinical and predominately caused by LPAIV strains. However, since around 2020 there has been increased global circulation of HPAI H5 viruses among wild birds, leading to substantial wild bird mortality and spillover events into poultry and mammals (4, 5). Wild bird reservoirs are a particular concern because they can spread HPAI to domestic poultry, and infections with LPAI H5 or H7 in poultry may evolve into highly pathogenic forms (2). The presence of alien (non-native) bird species, especially migratory, opportunistic or scavenging taxa, can disrupt local ecological balances and may influence the emergence and evolution of AI strains (6).

The African sacred ibis (*Threskiornis aethiopicus*), native to sub-Saharan Africa and limited parts of the Middle East, has established several breeding populations across Europe, with numbers increasing rapidly, and is currently listed as an invasive alien species of concern under EU Regulation 2016/1141 (7). The ever-expanding presence of the species has raised concerns about its potential impacts on local ecosystems and public health. In particular, *T. aethiopicus* has been suggested as a potential vector of various pathogens, including AIV, due to its migratory behavior and ecological interactions with waterfowl (8).

*T. aethiopicus* became invasive in Italy in recent decades, likely due to human-mediated introduction. The birds were initially imported for captivity, and kept in zoos, wildlife parks and even private collections where some were allowed free flight. Escape or release from captivity is supposed to be the primary origin of Italy’s feral populations (9, 10). The species established its first free-ranging breeding pair in Italy in 1989 at the Lame del Sesia Natural Park, Piedmont. Initially confined to north-western Italy, the population remained low until a rapid and exponential expansion began in the mid-2000s (11). By 2019, the wintering population in north-western Italy had reached over 10,800 individuals, with 1,249 nests recorded across 31 mixed-species colonies (10). The species now occurs throughout much of Northern Italy, with sporadic records in central regions and Sardinia, and is especially found in lowland areas with rice fields and wetlands, which represent the species’ preferred foraging habitats (10, 11). A National Management Plan, curated for Italy by the Italian Institute for Environmental Protection and Research (ISPRA) (11), outlines differentiated intervention strategies, which in Tuscany include local eradication and rapid response measures. The plan is primarily aimed at preventing further expansion of the African sacred ibis in the country.

In February 2025, a group of 20 African sacred ibises were culled in Tuscany (Central Italy) as part of the national control plan for this invasive species. The culled animals underwent necropsy and diagnostic investigations for public health monitoring activities, including PCR testing for avian influenza virus (AIV). Here, we report the detection of low pathogenic avian influenza (LPAI) virus subtype H5N2 in an African sacred ibis culled in Central Italy.

## 2 Method

### 2.1 Sample collection and processing

On February 21st 2025, a group of 20 African sacred ibises were culled as part of a national control plan for the species (11). The birds were grazing together in a cultivated field in Coltano, an administrative area of the province of Pisa (Tuscany, Central Italy; coordinates: 43°38′ N, 10°23′E). The carcasses were delivered to the Istituto Zooprofilattico Sperimentale del Lazio e della Toscana, Pisa, for public health monitoring activities as foreseen by the Tuscany Regional Health Surveillance Plan in Wild Fauna.

All subjects underwent necroscopic examination to assess the presence of external or internal gross lesions. Individual liver and intestinal content samples were tested for *Salmonella* spp. using culture medium, following the WOAH-recommended procedure (12). Pooled organ samples (kidney, spleen, heart, and brain) from each individual were tested for Usutu virus and West Nile virus (lineage 1 and 2) by real-time reverse transcription polymerase chain reaction (RT-PCR) (13), while brain tissue was tested for Newcastle Disease virus using RT-PCR (14).

### 2.2 Influenza A virus isolation and sequence analysis

Tracheal and cloacal swabs were collected from each of the 20 African sacred ibises, and subjected to RT-PCR (15, 16) for the detection of influenza A virus (IAV). For the single positive sample, confirmation and subtyping were performed at the European Union Reference Laboratory for Avian Influenza and Newcastle Disease. The partial genome (PA, HA, NP, NA, MP, and NS gene) of the positive AIV sample was obtained using the MinION device (Oxford Nanopore Technologies). The sample was amplified with two RT-PCR reactions, the first performed using CommonA-Uni12G (GCCGGAGCTCTGCAGATATCAGCGAAAGCAGG) and CommonA-Uni13G (GCCGGAGCTCTGCAGATATCAGTAGAAACAAGG) primers (17), while the second was performed using CommonA-Uni12 (GCCAGAGCTCTGCAGATATCAGCAAAAGCAGG) and CommonA-Uni13G primers, following the modified protocol from Van Poelvoorde et al. (17). The products obtained were pooled and purified using Agencourt AMPure XP beads. The library was prepared using Rapid sequencing V14—Amplicon sequencing SQK-RBK114.96 (Oxford Nanopore Technologies, Oxford, United Kingdom), loaded on a flowcell R10.4.1 and sequenced on MinION MK1C (Oxford Nanopore Technologies, Oxford, United Kingdom) for 6 h. Raw data was filtered using chopper v0.9.0 (18) with minimum quality of 10, minimum and maximum allowed length of 500 and 2,500, respectively. Surviving data was aligned against reference using minimap2 v2.17 (19, 20) and standard parameters. Consensus sequence was obtained by counting number of bases per position using samtools v1.6 (21) and selecting the most abundant base as the representative one, with a minimum coverage threshold of 100X fold coverage. The obtained sequences of the six gene segments were aligned using MAFFT v7 (22) and compared with the most closely related sequences identified using the Nucleotide Basic Local Alignment Search Tool (BLAST) in the GISAID EpiFlu^™^ database. Maximum Likelihood phylogenetic trees were obtained for each gene using IQTREE v1.6.6 (23, 24), with ultrafast bootstrap resampling (1,000 replications), and the software FigTree v1.4.4 was used to visualize the phylogenetic tree of each gene segment.^1^ Molecular analysis was performed using the open-source tool Flumut (25).

## 3 Results

Of the 20 examined African sacred ibises, necroscopic examination did not reveal any specific gross lesions, apart from gunshot wounds associated with the culling method. One specimen showed diffuse congestion of the coelomic viscera. One of the 20 subjects was ringed with an ISPRA ring; the ring was reported on the EURING portal, but no further information is available at the time of writing. *Salmonella* testing was negative in all liver and intestinal samples. RT-PCRs for Usutu virus, West Nile virus (lineage 1 and 2), and Newcastle Disease virus all returned negative results.

RT-PCR for IAV yielded a single positive result on both tracheal and cloacal swabs from one out of 20 sampled ibises. The positive subject was the same in which diffuse organ congestion had been observed. Subtyping identified a low pathogenic AIV of subtype H5N2. The sequences of six gene segments (PA, HA, NP, NA, MP, and NS gene) were compared with the most closely related sequences identified in the GISAID EpiFlu^™^ database (Supplementary File 1). The sequences were deposited in the GISAID EpiFlu^™^ database (accession number EPI_ISL_19906352) and in GenBank under the following accession numbers: PX136244 (PA), PX136245 (HA), PX136246 (NP), PX136247 (NA), PX136248 (MP), and PX136249 (NS), isolated from *A/sacred-ibis/Italy/25VIR4571-1/2025*. The topology of the phylogenetic trees of the six gene segments (Supplementary File 2) indicates that the virus clusters with Italian or other European LPAI viruses from 2023–2024 for the HA, NP, NA, MP, NS genes, while the PA gene clusters with LPAI viruses from Russia and Asia from 2021–2022. Molecular analysis (25) highlighted the presence of two mammalian adaptation markers in the HA protein (Table 1), namely “S155N” and the double mutation “S155N/T156A” (H5 numbering) (26). These markers were detected in all LPAI sequences within the analyzed dataset.

**Table 1:** Molecular markers identified in the analyzed virus.

Protein	Marker	Effect
HA	S155N	Increased virus binding to α2-6
	S155N/T156A	Increased virus binding to α2-6

## 4 Discussion

Despite its long presence in Europe, a definitive assessment of the ecological or sanitary status of the alien invasive species *T. aethiopicus* is lacking. Potential risks to autochthonous fauna include predation on amphibians, eggs or chicks of other bird species, and competition for breeding sites (9, 10, 27, 28). Health impacts on livestock remain poorly studied. The European Food Safety Authority (EFSA) and the European Centre for Disease Prevention and Control (ECDC) have identified the African sacred ibis in Italy as a species that should be carefully monitored, as it may act as a potential bridge for AIV transmission between wild birds and poultry (8). Several factors may contribute to this role, including the species’ growing numbers (10), known ecological interactions with waterfowl, proximity to poultry farms (29), and its migratory behavior, which involves high dispersal potential, especially among juvenile individuals (30).

In 2019, 12 faecal samples and 11 carcasses from a northern Italian colony were PCR-tested for AIV, all yielding negative results (31). Anyway, reliance mainly on environmental fecal material, the very small number of opportunistic carcasses, limited numbers from a single colony, and non-winter sampling windows, likely reduced detection probability. In France, Niquex et al. (32) detected antibodies against AIV subtypes H5 and N1 in African sacred ibises, suggesting previous exposure to AIV, although no evidence of active infection was found. The most recent EFSA report analyzing the highly pathogenic avian influenza (HPAI) epidemic of autumn–winter 2024–25 indicates that African sacred ibises may have introduced AIV to poultry in the Veneto region (8), as they were found to carry HPAI H5N1 viruses with a high degree of genetic similarity to those detected in fattening turkeys (Terregino, pers. comm.). Prior evidence of LPAI H7N1 AIV infections in asymptomatic sacred ibises was reported from South Africa in 2012 (33), with the species proposed as a bridge host for AIVs in ostrich farms, potentially facilitating transmission between wild and domestic birds (33, 34).

Wild birds in the orders Anseriformes and Charadriiformes are well-established reservoirs for both LPAI and HPAI strains (35), but the role of the African sacred ibis in AIV epidemiology remains poorly understood. The detection of LPAI H5N2 virus in *T. aethiopicus* in Central Italy by RT-PCR is not surprising, given a previous case of HPAI positivity in this species in Northern Italy (8) and its known ecological habits. However, this finding represents the first reported case of LPAIV in this alien species within the European context. Wetlands and croplands, such as those in Central Italy, may serve as critical interfaces for AIV transmission due to the congregation of migratory waterfowl and resident birds. African sacred ibises are indeed typically associated with wetland habitats. Nevertheless, the species has demonstrated high adaptability to anthropogenic environments, especially where natural habitats have been degraded, and is frequently observed at landfills or near poultry farms (30, 33, 35, 36). The known scavenging behavior of *T. aethiopicus* poses a particularly high risk for AIV exposure. Scavenging may facilitate pathogen transmission (37), potentially including ingestion of AIV-infected remains at landfills or in natural areas where carcass removal is delayed or not feasible (34, 38). Such behavior, combined with documented associations between sacred ibises and poultry farm outbreaks, supports their potential role as bridge-hosts linking wild AIV reservoirs to domestic flocks.

As the evidence presented in this study derives solely from RT-PCR and lacks virus isolation, infectivity assays, serology and tissue localization, the data do not allow determination of viral viability or replication competence, do not quantify shedding or transmissibility, and cannot establish clinical impact in this host; likewise, population-level implications cannot be inferred.

While LPAI H5N2 virus poses minimal direct zoonotic risk, its detection in an invasive species raises concerns about its potential to amplify viral spread to domestic poultry, particularly in Italy’s poultry-rich regions. H5 LPAI viruses may spontaneously evolve into high pathogenic forms, especially following infection in Galliformes birds (35). This potential for mutation, as documented in historical H5N2 outbreaks (39), underscores the need for continued vigilance, as recommended by the World Organization for Animal Health (1). The previous identification of HPAI A (H5N1) virus in *T. aethiopicus* in Northern Italy (8) is of additional concern, given their increasing presence in high-density poultry areas. Continued investigation into the role of the African sacred ibis and other invasive species as potential bridges between HPAI reservoirs and poultry remains essential. Follow-up studies, including virus isolation and, where feasible, serology and tissue localization in *T. aethiopicus* populations near poultry farms, will be important to clarify infection status, transmission potential and the species’ epidemiological role.

## 5 Conclusion

The presence of LPAI H5N2 virus in an alien African sacred ibis highlights the ecological and epidemiological risks posed by invasive species in AIV dynamics. Enforcing a culling plan for *T. aethiopicus* in Italy is critical to mitigate these risks. As a non-native species, it lacks natural population controls and may exacerbate, among other threats related to ecological or public and animal health issues, AIV transmission in wetland ecosystems, threatening both wild bird populations and the poultry industry. Targeted culling, combined with enhanced surveillance, could prevent the establishment of a novel reservoir, reducing the likelihood of viral spillover and mutation events. Such measures are essential to safeguard biodiversity, public health, and agricultural biosecurity, in areas where these birds are invasive.

## Data Availability

The datasets presented in this study can be found in online repositories. The names of the repository/repositories and accession number(s) can be found in the article/Supplementary material.

## Footnotes

^1^ http://tree.bio.ed.ac.uk/software/figtree/
