# Supplementary material for: Detection of avian influenza virus in the alien invasive African sacred ibis (Threskiornis aethiopicus) in Italy
Source: Front Vet Sci. 2025 Sep 8;12:1661089. doi: 10.3389/fvets.2025.1661089 (PMC12450672; doi:10.3389/fvets.2025.1661089)
Supplement: Supplementary file 1 [file Data_Sheet_1.PDF]

Supplementary File 1

Supplemental tables referring to the datasets of the six gene segments analyzed in this study. To view the EPI_SET IDs use the corresponding digital object identifier ("DOI"). Each supplemental Table contains relevant information referring to the sequences listed therein.

SUPPLEMENTAL TABLE

Data Availability

GISAID Identifier:	EPI_SET_250617pw
DOI:	https://doi.org/10.55876/gis8.250617pw

All genome sequences and associated metadata in this dataset are published in GISAID's EpiFlu database. To view the contributors of each individual sequence with details such as accession number, Virus name, Collection date, Originating Lab and Submitting Lab and the list of Authors, visit 10.55876/gis8.250107tk

Data Snapshot

EPI_SET_250617pw is composed of 155 individual viruses.
The collection dates range from 2018-11-15 to 2025-02-19;
Data were collected in 24 countries and territories.

SUPPLEMENTAL TABLE

Data Availability

GISAID Identifier:	EPI_SET_250617fo
DOI:	https://doi.org/10.55876/gis8.250617fo

All genome sequences and associated metadata in this dataset are published in GISAID's EpiFlu database. To view the contributors of each individual sequence with details such as accession number, Virus name, Collection date, Originating Lab and Submitting Lab and the list of Authors, visit 10.55876/gis8.250107tk

Data Snapshot

EPI_SET_250617fo is composed of 49 individual viruses.
The collection dates range from 2011-08-01 to 2024-10-16;
Data were collected in 14 countries and territories.

SUPPLEMENTAL TABLE

Data Availability

GISAID Identifier:	EPI_SET_250617bd
DOI:	https://doi.org/10.55876/gis8.250617bd

All genome sequences and associated metadata in this dataset are published in GISAID's EpiFlu database. To view the contributors of each individual sequence with details such as accession number, Virus name, Collection date, Originating Lab and Submitting Lab and the list of Authors, visit 10.55876/gis8.250107tk

Data Snapshot

EPI_SET_250617bd is composed of 50 individual viruses.
The collection dates range from 2015-10-26 to 2024-12-01;
Data were collected in 11 countries and territories.

SUPPLEMENTAL TABLE

Data Availability

GISAID Identifier:	EPI_SET_250617bh
DOI:	https://doi.org/10.55876/gis8.250617bh

All genome sequences and associated metadata in this dataset are published in GISAID's EpiFlu database. To view the contributors of each individual sequence with details such as accession number, Virus name, Collection date, Originating Lab and Submitting Lab and the list of Authors, visit 10.55876/gis8.250107tk

Data Snapshot

EPI_SET_250617bh is composed of 50 individual viruses.
The collection dates range from 2011-01-11 to 2025-03-02;
Data were collected in 12 countries and territories.

SUPPLEMENTAL TABLE

Data Availability

GISAID Identifier:	EPI_SET_250617rc
DOI:	https://doi.org/10.55876/gis8.250617rc

All genome sequences and associated metadata in this dataset are published in GISAID's EpiFlu database. To view the contributors of each individual sequence with details such as accession number, Virus name, Collection date, Originating Lab and Submitting Lab and the list of Authors, visit 10.55876/gis8.250107tk

Data Snapshot

EPI_SET_250617rc is composed of 50 individual viruses.
The collection dates range from 2014-11-24 to 2024-12-11;
Data were collected in 15 countries and territories.

SUPPLEMENTAL TABLE

Data Availability

GISAID Identifier:	EPI_SET_250617df
DOI:	https://doi.org/10.55876/gis8.250617df

All genome sequences and associated metadata in this dataset are published in GISAID's EpiFlu database. To view the contributors of each individual sequence with details such as accession number, Virus name, Collection date, Originating Lab and Submitting Lab and the list of Authors, visit 10.55876/gis8.250107tk

Data Snapshot

EPI_SET_250617df is composed of 106 individual viruses.
The collection dates range from 2010-11-23 to 2025-01-29;
Data were collected in 14 countries and territories.
