# Supplementary material for: Detection of avian influenza virus in the alien invasive African sacred ibis (Threskiornis aethiopicus) in Italy
Source: Front Vet Sci. 2025 Sep 8;12:1661089. doi: 10.3389/fvets.2025.1661089 (PMC12450672; doi:10.3389/fvets.2025.1661089)
Supplement: Supplementary file 2 [file Data_Sheet_2.PDF]

Supplementary File 2

Maximum likelihood phylogenetic tree of PA, HA, NP, NA, MP, NS genes. The H5N2 virus identified in the Sacred ibis is highlighted in blue. The trees were inferred using IQTREE v1.6.6. Ultrafast bootstrap supports higher than 80 are indicated next to the nodes.

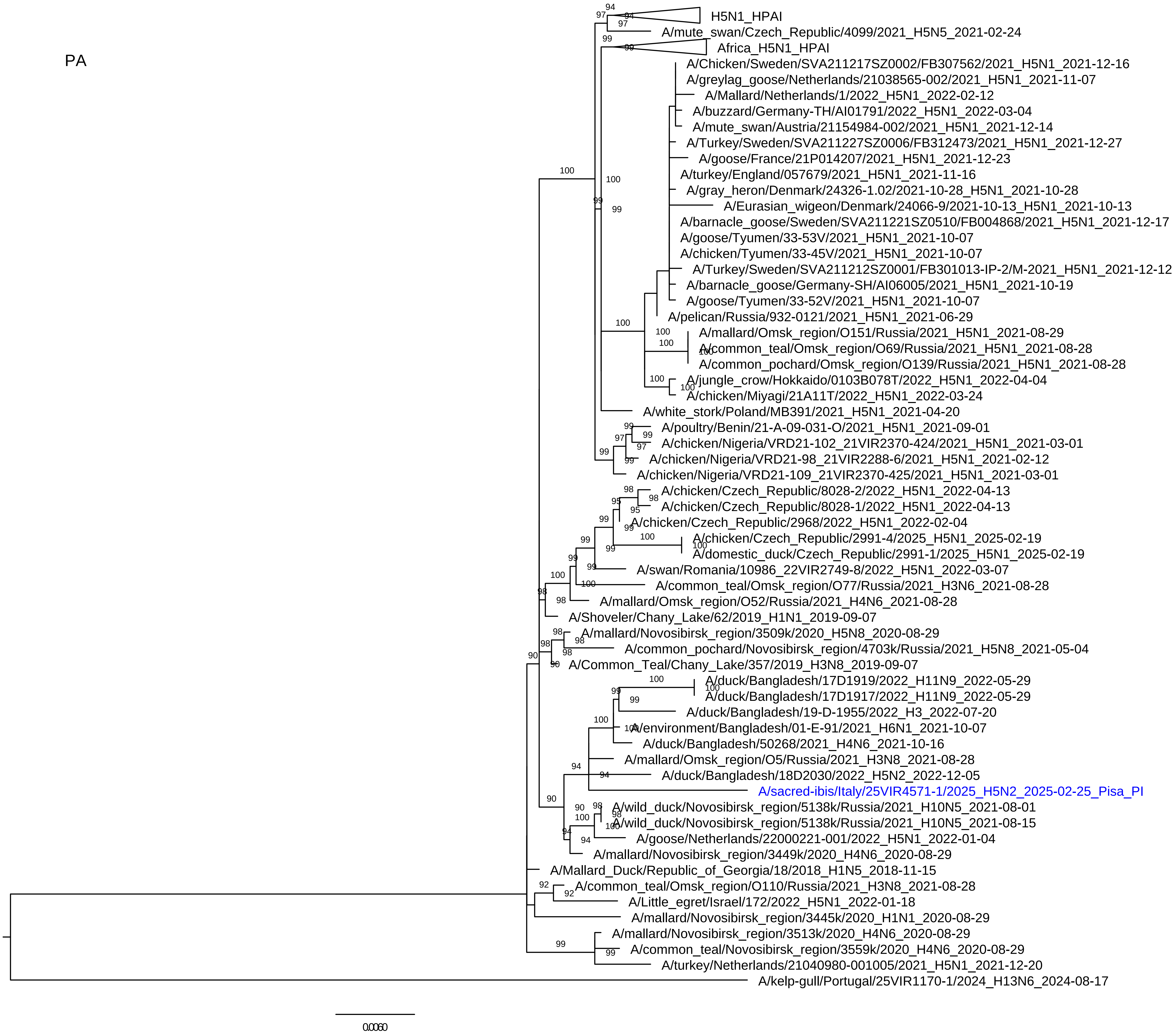
PA
H5N1_HPAI
A/mute_swan/Czech_Republic/4099/2021_H5N5_2021-02-24
Africa_H5N1_HPAI
A/Chicken/Sweden/SVA211217SZ0002/FB307562/2021_H5N1_2021-12-16
A/greylag_goose/Netherlands/21038565-002/2021_H5N1_2021-11-07
A/Mallard/Netherlands/1/2022_H5N1_2022-02-12
A/buzzard/Germany-TH/AI01791/2022_H5N1_2022-03-04
A/mute_swan/Austria/21154984-002/2021_H5N1_2021-12-14
A/Turkey/Sweden/SVA211227SZ0006/FB312473/2021_H5N1_2021-12-27
A/goose/France/21P014207/2021_H5N1_2021-12-23
A/turkey/England/057679/2021_H5N1_2021-11-16
A/gray_heron/Denmark/24326-1.02/2021-10-28_H5N1_2021-10-28
A/Eurasian_wigeon/Denmark/24066-9/2021-10-13_H5N1_2021-10-13
A/barnacle_goose/Sweden/SVA211221SZ0510/FB004868/2021_H5N1_2021-12-17
A/goose/Tyumen/33-53V/2021_H5N1_2021-10-07
A/chicken/Tyumen/33-45V/2021_H5N1_2021-10-07
A/Turkey/Sweden/SVA211212SZ0001/FB301013-IP-2/M-2021_H5N1_2021-12-12
A/barnacle_goose/Germany-SH/AI06005/2021_H5N1_2021-10-19
A/goose/Tyumen/33-52V/2021_H5N1_2021-10-07
A/pelican/Russia/932-0121/2021_H5N1_2021-06-29
A/mallard/Omsk_region/O151/Russia/2021_H5N1_2021-08-29
A/common_teal/Omsk_region/O69/Russia/2021_H5N1_2021-08-28
A/common_pochard/Omsk_region/O139/Russia/2021_H5N1_2021-08-28
A/jungle_crow/Hokkaido/0103B078T/2022_H5N1_2022-04-04
A/chicken/Miyagi/21A11T/2022_H5N1_2022-03-24
A/white_stork/Poland/MB391/2021_H5N1_2021-04-20
A/poultry/Benin/21-A-09-031-O/2021_H5N1_2021-09-01
A/chicken/Nigeria/VRD21-102_21VIR2370-424/2021_H5N1_2021-03-01
A/chicken/Nigeria/VRD21-98_21VIR2288-6/2021_H5N1_2021-02-12
A/chicken/Nigeria/VRD21-109_21VIR2370-425/2021_H5N1_2021-03-01
A/chicken/Czech_Republic/8028-2/2022_H5N1_2022-04-13
A/chicken/Czech_Republic/8028-1/2022_H5N1_2022-04-13
A/chicken/Czech_Republic/2968/2022_H5N1_2022-02-04
A/chicken/Czech_Republic/2991-4/2025_H5N1_2025-02-19
A/domestic_duck/Czech_Republic/2991-1/2025_H5N1_2025-02-19
A/swan/Romania/10986_22VIR2749-8/2022_H5N1_2022-03-07
A/common_teal/Omsk_region/O77/Russia/2021_H3N6_2021-08-28
A/mallard/Omsk_region/O52/Russia/2021_H4N6_2021-08-28
A/Shoveler/Chany_Lake/62/2019_H1N1_2019-09-07
A/mallard/Novosibirsk_region/3509k/2020_H5N8_2020-08-29
A/common_pochard/Novosibirsk_region/4703k/Russia/2021_H5N8_2021-05-04
A/Common_Teal/Chany_Lake/357/2019_H3N8_2019-09-07
A/duck/Bangladesh/17D1919/2022_H11N9_2022-05-29
A/duck/Bangladesh/17D1917/2022_H11N9_2022-05-29
A/duck/Bangladesh/19-D-1955/2022_H3_2022-07-20
A/environment/Bangladesh/01-E-91/2021_H6N1_2021-10-07
A/duck/Bangladesh/50268/2021_H4N6_2021-10-16
A/mallard/Omsk_region/O5/Russia/2021_H3N8_2021-08-28
A/duck/Bangladesh/18D2030/2022_H5N2_2022-12-05
A/sacred-ibis/Italy/25VIR4571-1/2025_H5N2_2025-02-25_Pisa_PI
A/wild_duck/Novosibirsk_region/5138k/Russia/2021_H10N5_2021-08-01
A/wild_duck/Novosibirsk_region/5138k/Russia/2021_H10N5_2021-08-15
A/goose/Netherlands/22000221-001/2022_H5N1_2022-01-04
A/mallard/Novosibirsk_region/3449k/2020_H4N6_2020-08-29
A/Mallard_Duck/Republic_of_Georgia/18/2018_H1N5_2018-11-15
A/common_teal/Omsk_region/O110/Russia/2021_H3N8_2021-08-28
A/Little_egret/Israel/172/2022_H5N1_2022-01-18
A/mallard/Novosibirsk_region/3445k/2020_H1N1_2020-08-29
A/mallard/Novosibirsk_region/3513k/2020_H4N6_2020-08-29
A/common_teal/Novosibirsk_region/3559k/2020_H4N6_2020-08-29
A/turkey/Netherlands/21040980-001005/2021_H5N1_2021-12-20
A/kelp-gull/Portugal/25VIR1170-1/2024_H13N6_2024-08-17
0.0060

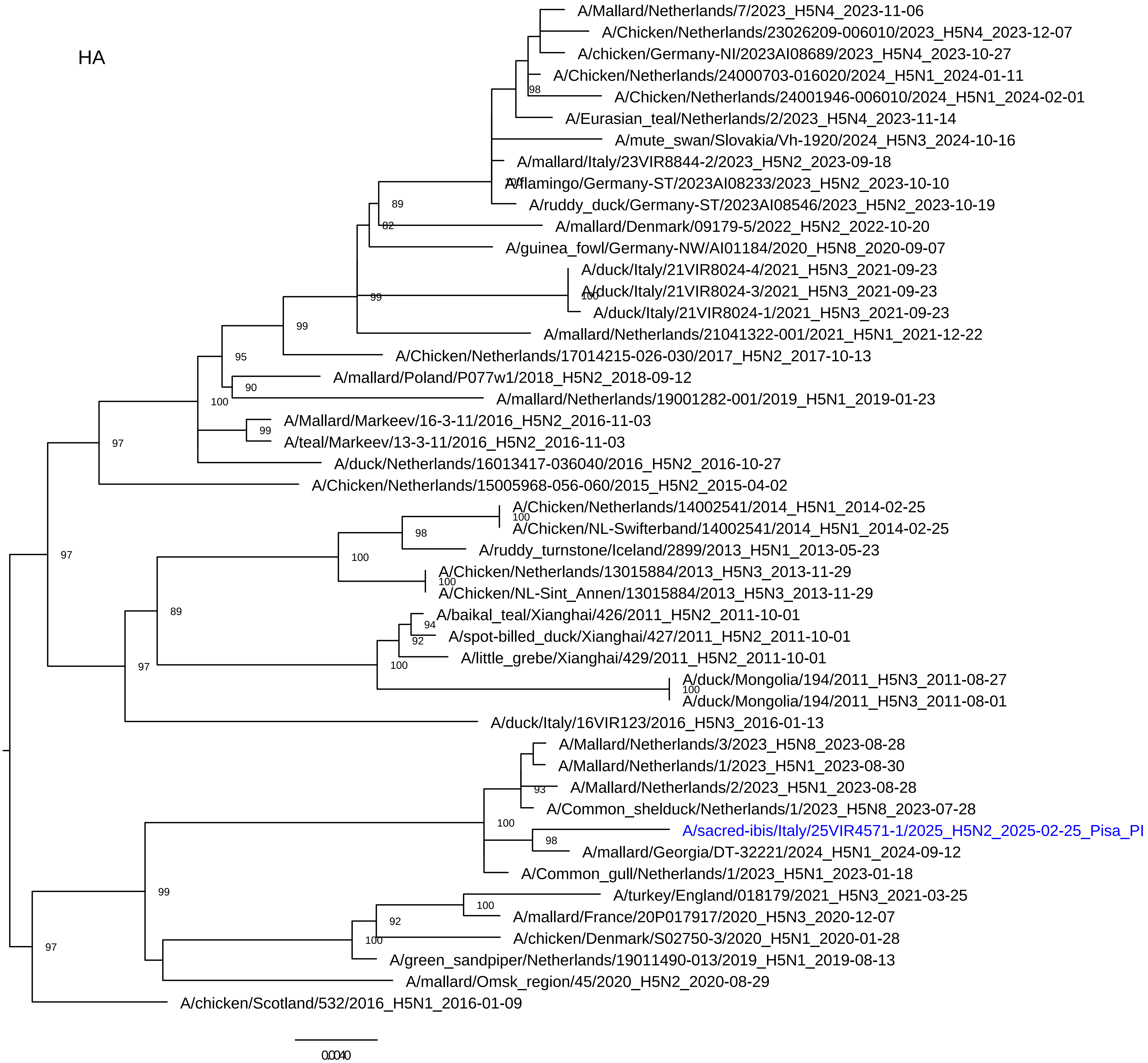
HA
A/Mallard/Netherlands/7/2023_H5N4_2023-11-06
A/Chicken/Netherlands/23026209-006010/2023_H5N4_2023-12-07
A/chicken/Germany-NI/2023AI08689/2023_H5N4_2023-10-27
A/Chicken/Netherlands/24000703-016020/2024_H5N1_2024-01-11
A/Chicken/Netherlands/24001946-006010/2024_H5N1_2024-02-01
A/Eurasian_teal/Netherlands/2/2023_H5N4_2023-11-14
A/mute_swan/Slovakia/Vh-1920/2024_H5N3_2024-10-16
A/mallard/Italy/23VIR8844-2/2023_H5N2_2023-09-18
A/flamingo/Germany-ST/2023AI08233/2023_H5N2_2023-10-10
A/ruddy_duck/Germany-ST/2023AI08546/2023_H5N2_2023-10-19
A/mallard/Denmark/09179-5/2022_H5N2_2022-10-20
A/guinea_fowl/Germany-NW/AI01184/2020_H5N8_2020-09-07
A/duck/Italy/21VIR8024-4/2021_H5N3_2021-09-23
A/duck/Italy/21VIR8024-3/2021_H5N3_2021-09-23
A/duck/Italy/21VIR8024-1/2021_H5N3_2021-09-23
A/mallard/Netherlands/21041322-001/2021_H5N1_2021-12-22
A/Chicken/Netherlands/17014215-026-030/2017_H5N2_2017-10-13
A/mallard/Poland/P077w1/2018_H5N2_2018-09-12
A/mallard/Netherlands/19001282-001/2019_H5N1_2019-01-23
A/Mallard/Markeev/16-3-11/2016_H5N2_2016-11-03
A/teal/Markeev/13-3-11/2016_H5N2_2016-11-03
A/duck/Netherlands/16013417-036040/2016_H5N2_2016-10-27
A/Chicken/Netherlands/15005968-056-060/2015_H5N2_2015-04-02
A/Chicken/Netherlands/14002541/2014_H5N1_2014-02-25
A/Chicken/NL-Swifterband/14002541/2014_H5N1_2014-02-25
A/ruddy_turnstone/Iceland/2899/2013_H5N1_2013-05-23
A/Chicken/Netherlands/13015884/2013_H5N3_2013-11-29
A/Chicken/NL-Sint_Annen/13015884/2013_H5N3_2013-11-29
A/baikal_teal/Xianghai/426/2011_H5N2_2011-10-01
A/spot-billed_duck/Xianghai/427/2011_H5N2_2011-10-01
A/little_grebe/Xianghai/429/2011_H5N2_2011-10-01
A/duck/Mongolia/194/2011_H5N3_2011-08-27
A/duck/Mongolia/194/2011_H5N3_2011-08-01
A/duck/Italy/16VIR123/2016_H5N3_2016-01-13
A/Mallard/Netherlands/3/2023_H5N8_2023-08-28
A/Mallard/Netherlands/1/2023_H5N1_2023-08-30
A/Mallard/Netherlands/2/2023_H5N1_2023-08-28
A/Common_shelduck/Netherlands/1/2023_H5N8_2023-07-28
A/sacred-ibis/Italy/25VIR4571-1/2025_H5N2_2025-02-25_Pisa_PI
A/mallard/Georgia/DT-32221/2024_H5N1_2024-09-12
A/Common_gull/Netherlands/1/2023_H5N1_2023-01-18
A/turkey/England/018179/2021_H5N3_2021-03-25
A/mallard/France/20P017917/2020_H5N3_2020-12-07
A/chicken/Denmark/S02750-3/2020_H5N1_2020-01-28
A/green_sandpiper/Netherlands/19011490-013/2019_H5N1_2019-08-13
A/mallard/Omsk_region/45/2020_H5N2_2020-08-29
A/chicken/Scotland/532/2016_H5N1_2016-01-09
98
100
89
82
99
100
99
95
90
100
99
97
100
98
100
100
97
89
94
92
100
100
97
93
100
98
99
100
92
100
97
0.0040

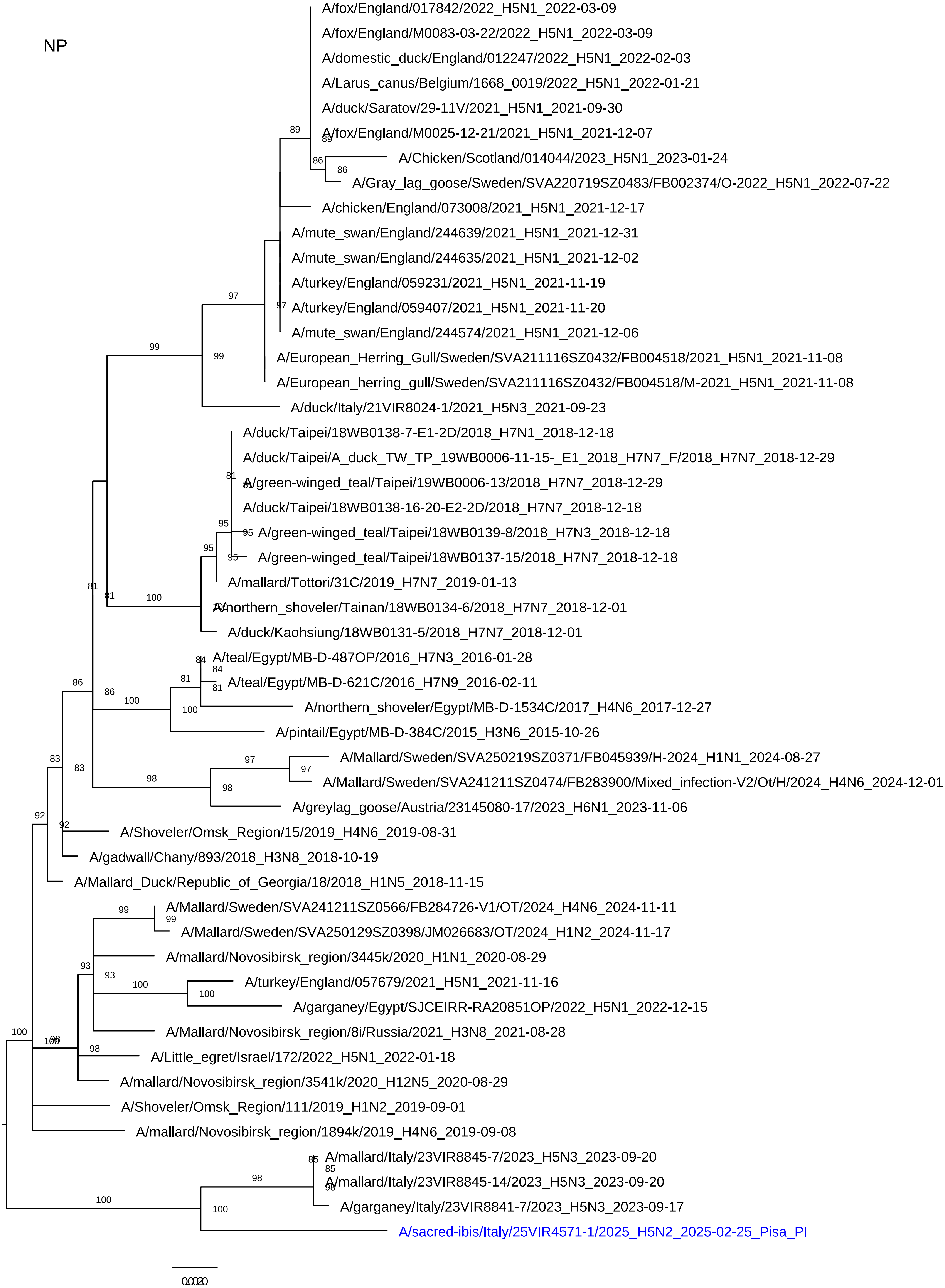
NP
A/fox/England/017842/2022_H5N1_2022-03-09
A/fox/England/M0083-03-22/2022_H5N1_2022-03-09
A/domestic_duck/England/012247/2022_H5N1_2022-02-03
A/Larus_canus/Belgium/1668_0019/2022_H5N1_2022-01-21
A/duck/Saratov/29-11V/2021_H5N1_2021-09-30
A/fox/England/M0025-12-21/2021_H5N1_2021-12-07
A/Chicken/Scotland/014044/2023_H5N1_2023-01-24
A/Gray_lag_goose/Sweden/SVA220719SZ0483/FB002374/O-2022_H5N1_2022-07-22
A/chicken/England/073008/2021_H5N1_2021-12-17
A/mute_swan/England/244639/2021_H5N1_2021-12-31
A/mute_swan/England/244635/2021_H5N1_2021-12-02
A/turkey/England/059231/2021_H5N1_2021-11-19
A/turkey/England/059407/2021_H5N1_2021-11-20
A/mute_swan/England/244574/2021_H5N1_2021-12-06
A/European_Herring_Gull/Sweden/SVA211116SZ0432/FB004518/2021_H5N1_2021-11-08
A/European_herring_gull/Sweden/SVA211116SZ0432/FB004518/M-2021_H5N1_2021-11-08
A/duck/Italy/21VIR8024-1/2021_H5N3_2021-09-23
A/duck/Taipei/18WB0138-7-E1-2D/2018_H7N1_2018-12-18
A/duck/Taipei/A_duck_TW_TP_19WB0006-11-15-_E1_2018_H7N7_F/2018_H7N7_2018-12-29
A/green-winged_teal/Taipei/19WB0006-13/2018_H7N7_2018-12-29
A/duck/Taipei/18WB0138-16-20-E2-2D/2018_H7N7_2018-12-18
A/green-winged_teal/Taipei/18WB0139-8/2018_H7N3_2018-12-18
A/green-winged_teal/Taipei/18WB0137-15/2018_H7N7_2018-12-18
A/mallard/Tottori/31C/2019_H7N7_2019-01-13
A/northern_shoveler/Tainan/18WB0134-6/2018_H7N7_2018-12-01
A/duck/Kaohsiung/18WB0131-5/2018_H7N7_2018-12-01
A/teal/Egypt/MB-D-487OP/2016_H7N3_2016-01-28
A/teal/Egypt/MB-D-621C/2016_H7N9_2016-02-11
A/northern_shoveler/Egypt/MB-D-1534C/2017_H4N6_2017-12-27
A/pintail/Egypt/MB-D-384C/2015_H3N6_2015-10-26
A/Mallard/Sweden/SVA250219SZ0371/FB045939/H-2024_H1N1_2024-08-27
A/Mallard/Sweden/SVA241211SZ0474/FB283900/Mixed_infection-V2/Ot/H/2024_H4N6_2024-12-01
A/greylag_goose/Austria/23145080-17/2023_H6N1_2023-11-06
A/Shoveler/Omsk_Region/15/2019_H4N6_2019-08-31
A/gadwall/Chany/893/2018_H3N8_2018-10-19
A/Mallard_Duck/Republic_of_Georgia/18/2018_H1N5_2018-11-15
A/Mallard/Sweden/SVA241211SZ0566/FB284726-V1/OT/2024_H4N6_2024-11-11
A/Mallard/Sweden/SVA250129SZ0398/JM026683/OT/2024_H1N2_2024-11-17
A/mallard/Novosibirsk_region/3445k/2020_H1N1_2020-08-29
A/turkey/England/057679/2021_H5N1_2021-11-16
A/garganey/Egypt/SJCEIRR-RA20851OP/2022_H5N1_2022-12-15
A/Mallard/Novosibirsk_region/8i/Russia/2021_H3N8_2021-08-28
A/Little_egret/Israel/172/2022_H5N1_2022-01-18
A/mallard/Novosibirsk_region/3541k/2020_H12N5_2020-08-29
A/Shoveler/Omsk_Region/111/2019_H1N2_2019-09-01
A/mallard/Novosibirsk_region/1894k/2019_H4N6_2019-09-08
A/mallard/Italy/23VIR8845-7/2023_H5N3_2023-09-20
A/mallard/Italy/23VIR8845-14/2023_H5N3_2023-09-20
A/garganey/Italy/23VIR8841-7/2023_H5N3_2023-09-17
A/sacred-ibis/Italy/25VIR4571-1/2025_H5N2_2025-02-25_Pisa_PI

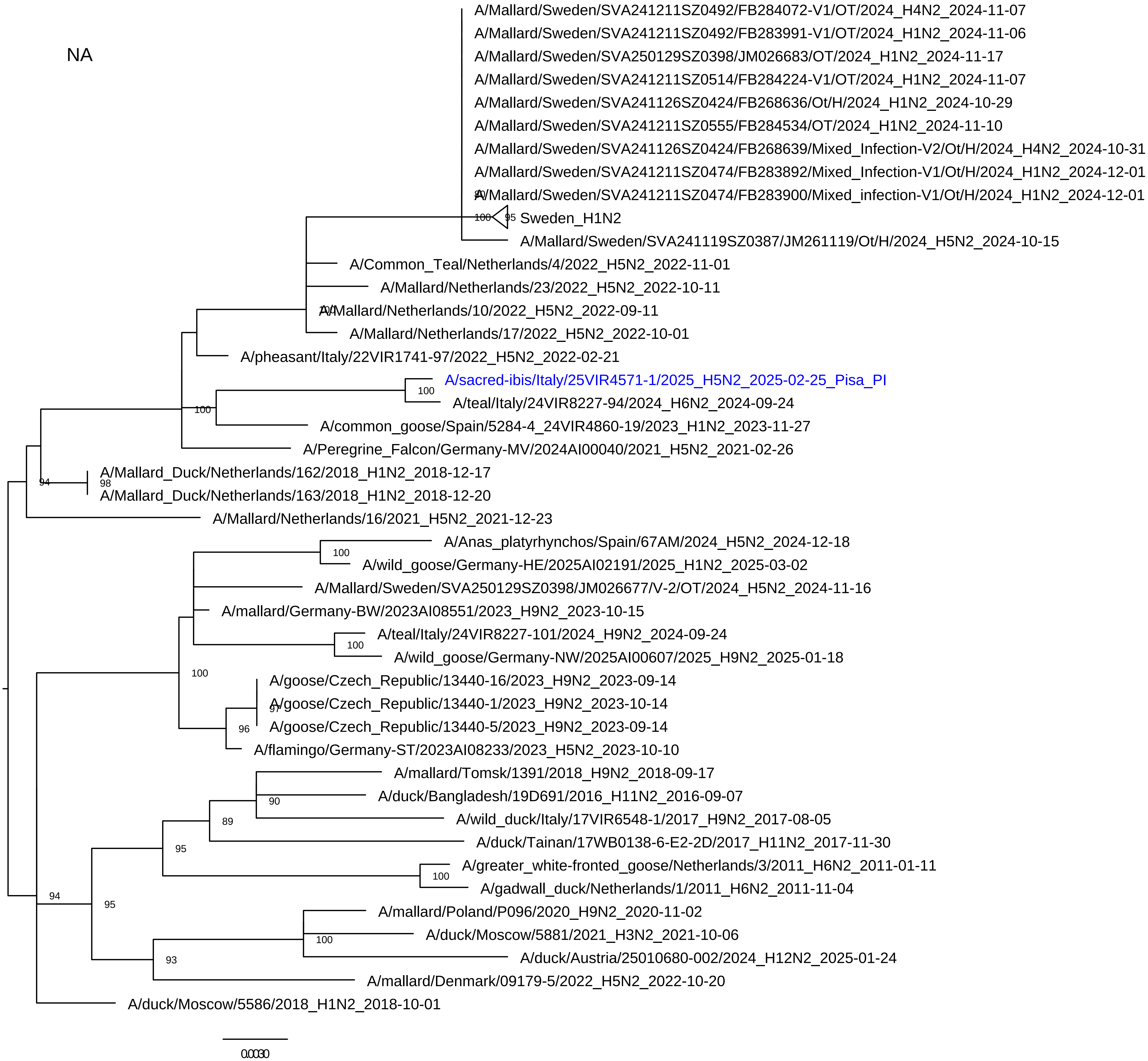
NA
A/Mallard/Sweden/SVA241211SZ0492/FB284072-V1/OT/2024_H4N2_2024-11-07
A/Mallard/Sweden/SVA241211SZ0492/FB283991-V1/OT/2024_H1N2_2024-11-06
A/Mallard/Sweden/SVA250129SZ0398/JM026683/OT/2024_H1N2_2024-11-17
A/Mallard/Sweden/SVA241211SZ0514/FB284224-V1/OT/2024_H1N2_2024-11-07
A/Mallard/Sweden/SVA241126SZ0424/FB268636/Ot/H/2024_H1N2_2024-10-29
A/Mallard/Sweden/SVA241211SZ0555/FB284534/OT/2024_H1N2_2024-11-10
A/Mallard/Sweden/SVA241126SZ0424/FB268639/Mixed_Infection-V2/Ot/H/2024_H4N2_2024-10-31
A/Mallard/Sweden/SVA241211SZ0474/FB283892/Mixed_Infection-V1/Ot/H/2024_H1N2_2024-12-01
A/Mallard/Sweden/SVA241211SZ0474/FB283900/Mixed_infection-V1/Ot/H/2024_H1N2_2024-12-01
100
95
Sweden_H1N2
A/Mallard/Sweden/SVA241119SZ0387/JM261119/Ot/H/2024_H5N2_2024-10-15
A/Common_Teal/Netherlands/4/2022_H5N2_2022-11-01
A/Mallard/Netherlands/23/2022_H5N2_2022-10-11
A/Mallard/Netherlands/10/2022_H5N2_2022-09-11
A/Mallard/Netherlands/17/2022_H5N2_2022-10-01
A/pheasant/Italy/22VIR1741-97/2022_H5N2_2022-02-21
A/sacred-ibis/Italy/25VIR4571-1/2025_H5N2_2025-02-25_Pisa_PI
100
A/teal/Italy/24VIR8227-94/2024_H6N2_2024-09-24
100
A/common_goose/Spain/5284-4_24VIR4860-19/2023_H1N2_2023-11-27
A/Peregrine_Falcon/Germany-MV/2024AI00040/2021_H5N2_2021-02-26
A/Mallard_Duck/Netherlands/162/2018_H1N2_2018-12-17
94
98
A/Mallard_Duck/Netherlands/163/2018_H1N2_2018-12-20
A/Mallard/Netherlands/16/2021_H5N2_2021-12-23
A/Anas_platyrhynchos/Spain/67AM/2024_H5N2_2024-12-18
100
A/wild_goose/Germany-HE/2025AI02191/2025_H1N2_2025-03-02
A/Mallard/Sweden/SVA250129SZ0398/JM026677/V-2/OT/2024_H5N2_2024-11-16
A/mallard/Germany-BW/2023AI08551/2023_H9N2_2023-10-15
A/teal/Italy/24VIR8227-101/2024_H9N2_2024-09-24
100
A/wild_goose/Germany-NW/2025AI00607/2025_H9N2_2025-01-18
100
A/goose/Czech_Republic/13440-16/2023_H9N2_2023-09-14
A/goose/Czech_Republic/13440-1/2023_H9N2_2023-10-14
96
A/goose/Czech_Republic/13440-5/2023_H9N2_2023-09-14
A/flamingo/Germany-ST/2023AI08233/2023_H5N2_2023-10-10
A/mallard/Tomsk/1391/2018_H9N2_2018-09-17
A/duck/Bangladesh/19D691/2016_H11N2_2016-09-07
90
89
A/wild_duck/Italy/17VIR6548-1/2017_H9N2_2017-08-05
A/duck/Tainan/17WB0138-6-E2-2D/2017_H11N2_2017-11-30
95
A/greater_white-fronted_goose/Netherlands/3/2011_H6N2_2011-01-11
100
A/gadwall_duck/Netherlands/1/2011_H6N2_2011-11-04
94
95
A/mallard/Poland/P096/2020_H9N2_2020-11-02
A/duck/Moscow/5881/2021_H3N2_2021-10-06
100
A/duck/Austria/25010680-002/2024_H12N2_2025-01-24
93
A/mallard/Denmark/09179-5/2022_H5N2_2022-10-20
A/duck/Moscow/5586/2018_H1N2_2018-10-01

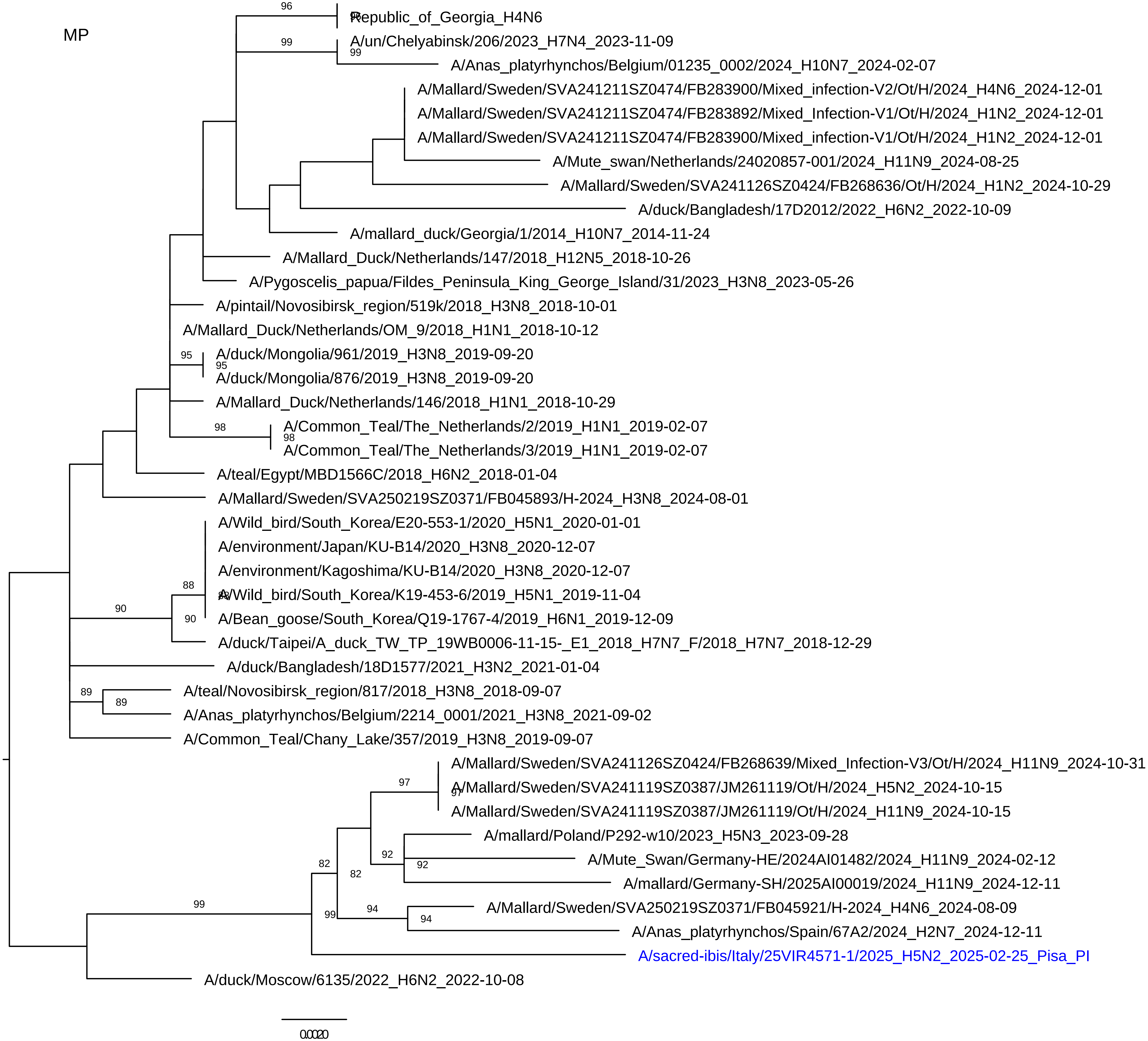
MP
Republic_of_Georgia_H4N6
A/un/Chelyabinsk/206/2023_H7N4_2023-11-09
A/Anas_platyrhynchos/Belgium/01235_0002/2024_H10N7_2024-02-07
A/Mallard/Sweden/SVA241211SZ0474/FB283900/Mixed_infection-V2/Ot/H/2024_H4N6_2024-12-01
A/Mallard/Sweden/SVA241211SZ0474/FB283892/Mixed_Infection-V1/Ot/H/2024_H1N2_2024-12-01
A/Mallard/Sweden/SVA241211SZ0474/FB283900/Mixed_infection-V1/Ot/H/2024_H1N2_2024-12-01
A/Mute_swan/Netherlands/24020857-001/2024_H11N9_2024-08-25
A/Mallard/Sweden/SVA241126SZ0424/FB268636/Ot/H/2024_H1N2_2024-10-29
A/duck/Bangladesh/17D2012/2022_H6N2_2022-10-09
A/mallard_duck/Georgia/1/2014_H10N7_2014-11-24
A/Mallard_Duck/Netherlands/147/2018_H12N5_2018-10-26
A/Pygoscelis_papua/Fildes_Peninsula_King_George_Island/31/2023_H3N8_2023-05-26
A/pintail/Novosibirsk_region/519k/2018_H3N8_2018-10-01
A/Mallard_Duck/Netherlands/OM_9/2018_H1N1_2018-10-12
A/duck/Mongolia/961/2019_H3N8_2019-09-20
A/duck/Mongolia/876/2019_H3N8_2019-09-20
A/Mallard_Duck/Netherlands/146/2018_H1N1_2018-10-29
A/Common_Teal/The_Netherlands/2/2019_H1N1_2019-02-07
A/Common_Teal/The_Netherlands/3/2019_H1N1_2019-02-07
A/teal/Egypt/MBD1566C/2018_H6N2_2018-01-04
A/Mallard/Sweden/SVA250219SZ0371/FB045893/H-2024_H3N8_2024-08-01
A/Wild_bird/South_Korea/E20-553-1/2020_H5N1_2020-01-01
A/environment/Japan/KU-B14/2020_H3N8_2020-12-07
A/environment/Kagoshima/KU-B14/2020_H3N8_2020-12-07
A/Wild_bird/South_Korea/K19-453-6/2019_H5N1_2019-11-04
A/Bean_goose/South_Korea/Q19-1767-4/2019_H6N1_2019-12-09
A/duck/Taipei/A_duck_TW_TP_19WB0006-11-15-_E1_2018_H7N7_F/2018_H7N7_2018-12-29
A/duck/Bangladesh/18D1577/2021_H3N2_2021-01-04
A/teal/Novosibirsk_region/817/2018_H3N8_2018-09-07
A/Anas_platyrhynchos/Belgium/2214_0001/2021_H3N8_2021-09-02
A/Common_Teal/Chany_Lake/357/2019_H3N8_2019-09-07
A/Mallard/Sweden/SVA241126SZ0424/FB268639/Mixed_Infection-V3/Ot/H/2024_H11N9_2024-10-31
A/Mallard/Sweden/SVA241119SZ0387/JM261119/Ot/H/2024_H5N2_2024-10-15
A/Mallard/Sweden/SVA241119SZ0387/JM261119/Ot/H/2024_H11N9_2024-10-15
A/mallard/Poland/P292-w10/2023_H5N3_2023-09-28
A/Mute_Swan/Germany-HE/2024AI01482/2024_H11N9_2024-02-12
A/mallard/Germany-SH/2025AI00019/2024_H11N9_2024-12-11
A/Mallard/Sweden/SVA250219SZ0371/FB045921/H-2024_H4N6_2024-08-09
A/Anas_platyrhynchos/Spain/67A2/2024_H2N7_2024-12-11
A/sacred-ibis/Italy/25VIR4571-1/2025_H5N2_2025-02-25_Pisa_PI
A/duck/Moscow/6135/2022_H6N2_2022-10-08
96
99
99
95
95
98
98
88
90
90
89
89
97
92
92
82
82
99
99
94
94
0.020

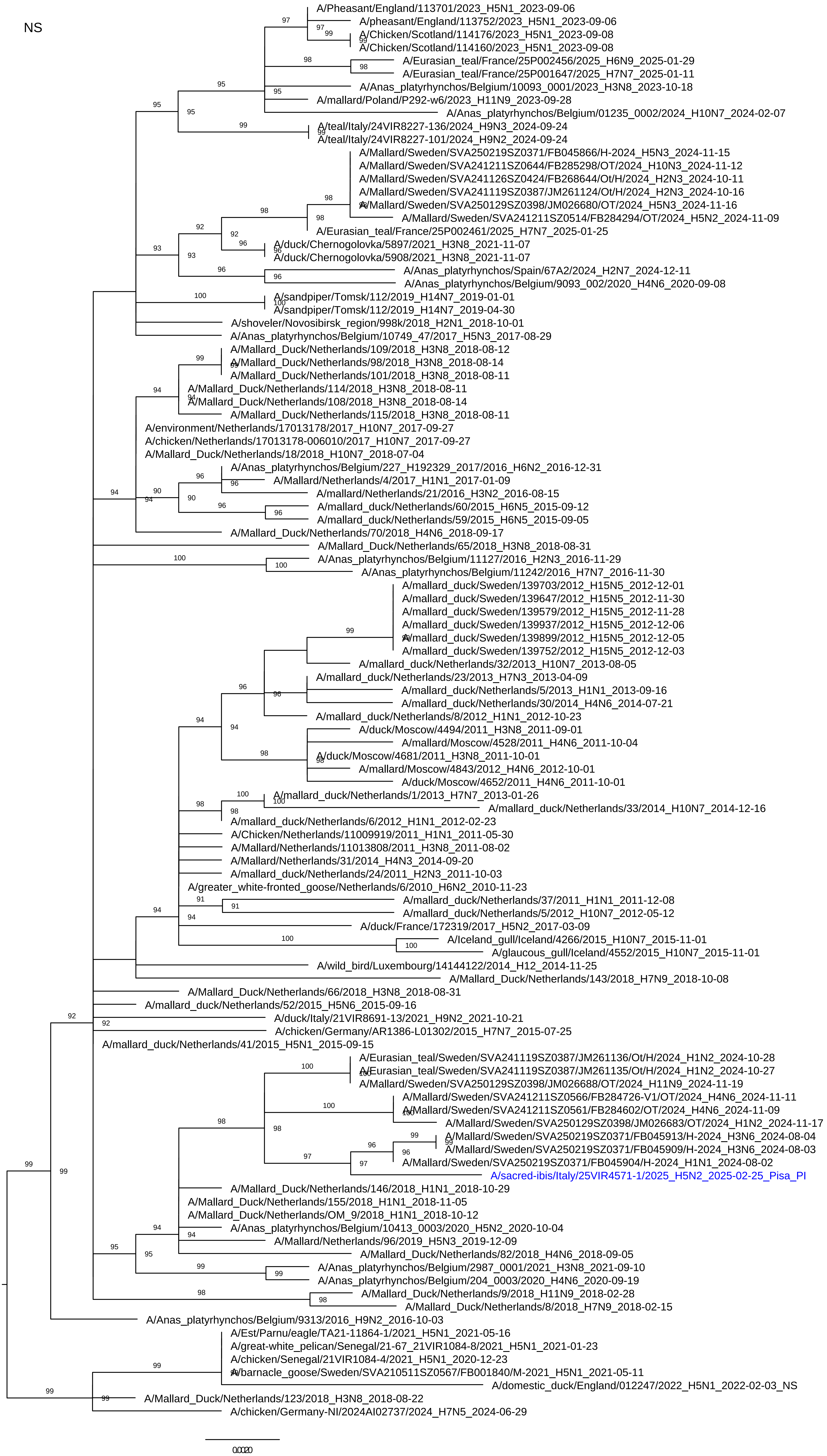
NS
A/Pheasant/England/113701/2023_H5N1_2023-09-06
A/pheasant/England/113752/2023_H5N1_2023-09-06
A/Chicken/Scotland/114176/2023_H5N1_2023-09-08
A/Chicken/Scotland/114160/2023_H5N1_2023-09-08
A/Eurasian_teal/France/25P002456/2025_H6N9_2025-01-29
A/Eurasian_teal/France/25P001647/2025_H7N7_2025-01-11
A/Anas_platyrhynchos/Belgium/10093_0001/2023_H3N8_2023-10-18
A/mallard/Poland/P292-w6/2023_H11N9_2023-09-28
A/Anas_platyrhynchos/Belgium/01235_0002/2024_H10N7_2024-02-07
A/teal/Italy/24VIR8227-136/2024_H9N3_2024-09-24
A/teal/Italy/24VIR8227-101/2024_H9N2_2024-09-24
A/Mallard/Sweden/SVA250219SZ0371/FB045866/H-2024_H5N3_2024-11-15
A/Mallard/Sweden/SVA241211SZ0644/FB285298/OT/2024_H10N3_2024-11-12
A/Mallard/Sweden/SVA241126SZ0424/FB268644/Ot/H/2024_H2N3_2024-10-11
A/Mallard/Sweden/SVA241119SZ0387/JM261124/Ot/H/2024_H2N3_2024-10-16
A/Mallard/Sweden/SVA250129SZ0398/JM026680/OT/2024_H5N3_2024-11-16
A/Mallard/Sweden/SVA241211SZ0514/FB284294/OT/2024_H5N2_2024-11-09
A/Eurasian_teal/France/25P002461/2025_H7N7_2025-01-25
A/duck/Chernogolovka/5897/2021_H3N8_2021-11-07
A/duck/Chernogolovka/5908/2021_H3N8_2021-11-07
A/Anas_platyrhynchos/Spain/67A2/2024_H2N7_2024-12-11
A/Anas_platyrhynchos/Belgium/9093_002/2020_H4N6_2020-09-08
A/sandpiper/Tomsk/112/2019_H14N7_2019-01-01
A/sandpiper/Tomsk/112/2019_H14N7_2019-04-30
A/shoveler/Novosibirsk_region/998k/2018_H2N1_2018-10-01
A/Anas_platyrhynchos/Belgium/10749_47/2017_H5N3_2017-08-29
A/Mallard_Duck/Netherlands/109/2018_H3N8_2018-08-12
A/Mallard_Duck/Netherlands/98/2018_H3N8_2018-08-14
A/Mallard_Duck/Netherlands/101/2018_H3N8_2018-08-11
A/Mallard_Duck/Netherlands/114/2018_H3N8_2018-08-11
A/Mallard_Duck/Netherlands/108/2018_H3N8_2018-08-14
A/Mallard_Duck/Netherlands/115/2018_H3N8_2018-08-11
A/environment/Netherlands/17013178/2017_H10N7_2017-09-27
A/chicken/Netherlands/17013178-006010/2017_H10N7_2017-09-27
A/Mallard_Duck/Netherlands/18/2018_H10N7_2018-07-04
A/Anas_platyrhynchos/Belgium/227_H192329_2017/2016_H6N2_2016-12-31
A/Mallard/Netherlands/4/2017_H1N1_2017-01-09
A/mallard/Netherlands/21/2016_H3N2_2016-08-15
A/mallard_duck/Netherlands/60/2015_H6N5_2015-09-12
A/mallard_duck/Netherlands/59/2015_H6N5_2015-09-05
A/Mallard_Duck/Netherlands/70/2018_H4N6_2018-09-17
A/Mallard_Duck/Netherlands/65/2018_H3N8_2018-08-31
A/Anas_platyrhynchos/Belgium/11127/2016_H2N3_2016-11-29
A/Anas_platyrhynchos/Belgium/11242/2016_H7N7_2016-11-30
A/mallard_duck/Sweden/139703/2012_H15N5_2012-12-01
A/mallard_duck/Sweden/139647/2012_H15N5_2012-11-30
A/mallard_duck/Sweden/139579/2012_H15N5_2012-11-28
A/mallard_duck/Sweden/139937/2012_H15N5_2012-12-06
A/mallard_duck/Sweden/139899/2012_H15N5_2012-12-05
A/mallard_duck/Sweden/139752/2012_H15N5_2012-12-03
A/mallard_duck/Netherlands/32/2013_H10N7_2013-08-05
A/mallard_duck/Netherlands/23/2013_H7N3_2013-04-09
A/mallard_duck/Netherlands/5/2013_H1N1_2013-09-16
A/mallard_duck/Netherlands/30/2014_H4N6_2014-07-21
A/mallard_duck/Netherlands/8/2012_H1N1_2012-10-23
A/duck/Moscow/4494/2011_H3N8_2011-09-01
A/mallard/Moscow/4528/2011_H4N6_2011-10-04
A/duck/Moscow/4681/2011_H3N8_2011-10-01
A/mallard/Moscow/4843/2012_H4N6_2012-10-01
A/duck/Moscow/4652/2011_H4N6_2011-10-01
A/mallard_duck/Netherlands/1/2013_H7N7_2013-01-26
A/mallard_duck/Netherlands/33/2014_H10N7_2014-12-16
A/mallard_duck/Netherlands/6/2012_H1N1_2012-02-23
A/Chicken/Netherlands/11009919/2011_H1N1_2011-05-30
A/Mallard/Netherlands/11013808/2011_H3N8_2011-08-02
A/Mallard/Netherlands/31/2014_H4N3_2014-09-20
A/mallard_duck/Netherlands/24/2011_H2N3_2011-10-03
A/greater_white-fronted_goose/Netherlands/6/2010_H6N2_2010-11-23
A/mallard_duck/Netherlands/37/2011_H1N1_2011-12-08
A/mallard_duck/Netherlands/5/2012_H10N7_2012-05-12
A/duck/France/172319/2017_H5N2_2017-03-09
A/Iceland_gull/Iceland/4266/2015_H10N7_2015-11-01
A/glaucous_gull/Iceland/4552/2015_H10N7_2015-11-01
A/wild_bird/Luxembourg/14144122/2014_H12_2014-11-25
A/Mallard_Duck/Netherlands/143/2018_H7N9_2018-10-08
A/Mallard_Duck/Netherlands/66/2018_H3N8_2018-08-31
A/mallard_duck/Netherlands/52/2015_H5N6_2015-09-16
A/duck/Italy/21VIR8691-13/2021_H9N2_2021-10-21
A/chicken/Germany/AR1386-L01302/2015_H7N7_2015-07-25
A/mallard_duck/Netherlands/41/2015_H5N1_2015-09-15
A/Eurasian_teal/Sweden/SVA241119SZ0387/JM261136/Ot/H/2024_H1N2_2024-10-28
A/Eurasian_teal/Sweden/SVA241119SZ0387/JM261135/Ot/H/2024_H1N2_2024-10-27
A/Mallard/Sweden/SVA250129SZ0398/JM026688/OT/2024_H11N9_2024-11-19
A/Mallard/Sweden/SVA241211SZ0566/FB284726-V1/OT/2024_H4N6_2024-11-11
A/Mallard/Sweden/SVA241211SZ0561/FB284602/OT/2024_H4N6_2024-11-09
A/Mallard/Sweden/SVA250129SZ0398/JM026683/OT/2024_H1N2_2024-11-17
A/Mallard/Sweden/SVA250219SZ0371/FB045913/H-2024_H3N6_2024-08-04
A/Mallard/Sweden/SVA250219SZ0371/FB045909/H-2024_H3N6_2024-08-03
A/Mallard/Sweden/SVA250219SZ0371/FB045904/H-2024_H1N1_2024-08-02
A/sacred-ibis/Italy/25VIR4571-1/2025_H5N2_2025-02-25_Pisa_PI
A/Mallard_Duck/Netherlands/146/2018_H1N1_2018-10-29
A/Mallard_Duck/Netherlands/155/2018_H1N1_2018-11-05
A/Mallard_Duck/Netherlands/OM_9/2018_H1N1_2018-10-12
A/Anas_platyrhynchos/Belgium/10413_0003/2020_H5N2_2020-10-04
A/Mallard/Netherlands/96/2019_H5N3_2019-12-09
A/Mallard_Duck/Netherlands/82/2018_H4N6_2018-09-05
A/Anas_platyrhynchos/Belgium/2987_0001/2021_H3N8_2021-09-10
A/Anas_platyrhynchos/Belgium/204_0003/2020_H4N6_2020-09-19
A/Mallard_Duck/Netherlands/9/2018_H11N9_2018-02-28
A/Mallard_Duck/Netherlands/8/2018_H7N9_2018-02-15
A/Anas_platyrhynchos/Belgium/9313/2016_H9N2_2016-10-03
A/Est/Parnu/eagle/TA21-11864-1/2021_H5N1_2021-05-16
A/great-white_pelican/Senegal/21-67_21VIR1084-8/2021_H5N1_2021-01-23
A/chicken/Senegal/21VIR1084-4/2021_H5N1_2020-12-23
A/barnacle_goose/Sweden/SVA210511SZ0567/FB001840/M-2021_H5N1_2021-05-11
A/domestic_duck/England/012247/2022_H5N1_2022-02-03_NS
A/Mallard_Duck/Netherlands/123/2018_H3N8_2018-08-22
A/chicken/Germany-NI/2024AI02737/2024_H7N5_2024-06-29
